# Vancomycin Serum Concentration after 48 h of Administration: A 3-Years Survey in an Intensive Care Unit

**DOI:** 10.3390/antibiotics9110793

**Published:** 2020-11-10

**Authors:** Nicolas Perin, Claire Roger, Grégory Marin, Nicolas Molinari, Alexandre Evrard, Jean-Philippe Lavigne, Saber Barbar, Pierre Géraud Claret, Caroline Boutin, Laurent Muller, Jeffrey Lipman, Jean-Yves Lefrant, Samir Jaber, Jason A. Roberts

**Affiliations:** 1 Service des Réanimations, Pôle Anesthésie Réanimation Douleur Urgence, CHU Nîmes, 30029 Nîmes, France; claire.roger@chu-nimes.fr (C.R.); saber.barbar@chu-nimes.fr (S.B.); pierre.geraud.claret@chu-nimes.fr (P.G.C.); caroline.boutin@chu-nimes.fr (C.B.); laurent.muller@chu-nimes.fr (L.M.); j.lipman@uq.edu.au (J.L.); jean.yves.lefrant@chu-nimes.fr (J.-Y.L.); j.roberts2@uq.edu.au (J.A.R.); 2 Equipe D’accueil 2992 Caractéristiques Féminines des Interfaces Vasculaires, Faculté de Médecine, Université de Montpellier, 34090 Montpellier, France; 3 IMAG, CNRS, Université de Montpellier, Department of Statistics, CHU Montpellier, 34295 Montpellier, France; g-marin@chu-montpellier.fr (G.M.); n-molinari@chu-montpellier.fr (N.M.); 4 Laboratoire de Biochimie, Centre Hospitalier Universitaire (CHU) de Nîmes, Hôpital Carémeau, 30029 Nîmes, France; alexandre.EVRARD@chu-nimes.fr; 5 VBMI, INSERM U1047, Université de Montpellier, Laboratoire de Microbiologie, CHU de Nîmes, 30029 Nîmes, France; jean.philippe.lavigne@chu-nimes.fr; 6 Department of Intensive Care Medicine, Royal Brisbane and Womens’ Hospital, Brisbane 4029, QLD, Australia; 7 UQ Centre for Clinical Research, The University of Queensland, Brisbane 4029, QLD, Australia; 8 Département d’Anesthésie Réanimation B, Saint Eloi ICU, Montpellier University Hospital, 34295 Montpellier, France; s-jaber@chu-montpellier.fr; 9 Centre for Translational Anti-Infective Pharmacodynamics, School of Pharmacy, The University of Queensland, Brisbane 4029, QLD, Australia; 10 Pharmacy Department, Royal Brisbane and Womens’ Hospital, Brisbane 4029, QLD, Australia

**Keywords:** intensive care, infection, vancomycin, pharmacokinetic, drug monitoring

## Abstract

The present study assessed the proportion of intensive care unit (ICU) patients who had a vancomycin serum concentration between 20 and 25 mg/L after 24–48 h of intravenous vancomycin administration. From 2016 to 2018, adult ICU patients with vancomycin continuous infusion (CI) for any indication were included. The primary outcome was the proportion of patients with a first-available vancomycin serum concentration between 20–25 mg/L at 24 h (D2) or 48 h (D3). Of 3894 admitted ICU patients, 179 were included. A median loading dose of 15.6 (interquartile range (IQR) = (12.5–20.8) mg/kg) was given in 151/179 patients (84%). The median daily doses of vancomycin infusion for D1 and D2 were 2000 [(IQR (1600–2000)) and 2000 (IQR (2000–2500)) mg/d], respectively. The median duration of treatment was 4 (2–7) days. At D2 or D3, the median value of first serum vancomycin concentration was 19.8 (IQR (16.0–25.1)) with serum vancomycin concentration between 20–25 mg/L reported in 43 patients (24%). Time spent in the ICU before vancomycin initiation was the only risk factor of non-therapeutic concentration at D2 or D3. Acute kidney injury occurred significantly more when vancomycin concentration was supra therapeutic at D2 or D3. At D28, 44 (26%) patients had died. These results emphasize the need of appropriate loading dose and regular monitoring to improve vancomycin efficacy and avoid renal toxicity.

## 1. Introduction

Septic shock is one of the main causes of admission into intensive care units (ICUs) with a related mortality rate of approximately 40% [1]. Among gram-positive pathogens, methicillin-resistant *Staphylococcus aureus* (MRSA) is a risk factor for mortality [2]. As early administration of adequate doses of effective antibiotics is a priority in the management of infected ICU patients, vancomycin remains a cornerstone choice in antibiotic treatment, particularly in the context of potentially resistant Gram-positive pathogens [3]. The risk of suboptimal drug dosing has led to recommendations for high vancomycin dosing coupled with therapeutic drug monitoring (TDM) for critically ill patients [4,5].

Vancomycin’s pharmacodynamics are best described using the area under the curve to minimum inhibitory concentration (AUC/MIC) ratio, with a value >400 recommended to ensure good patient outcome (cure of infection and avoidance of mortality) [6,7,8]. In clinical practice, because of difficulties in establishing the AUC/MIC at bedside, French guidelines and the previous 2009 American guidelines recommended a trough of 15–20 mg/L in serious patients when vancomycin was given by intermittent infusion [5,9]. In the new 2020 American guidelines, published after the completion of this study, only a target based on AUC is recommended when using an intermittent infusion [8]. However, continuous infusion (CI) has been advocated, even though only one study has reported a better patient outcome [10]. The arguments in favour of continuous infusions are a faster attainment of target concentrations, less variability in serum vancomycin concentrations, and less nephrotoxicity [11,12,13]. Moreover, it is cheaper and logistically easier to complete TDM and AUC calculations [14]. Hence, the 2020 American guidelines recommend a trough of 20–25 mg/L in serious patients when vancomycin is given by continuous infusion [8].

Recent studies reported that target serum concentrations were not reached in 30 to 50% of ICU patients given a vancomycin CI infusion [15,16,17]. These findings could be explained by the alterations in vancomycin pharmacokinetics in ICU patients, especially in patients with an augmented renal clearance (ARC) >130 mL/min [18,19]. Because the inflammatory response leads to an increased volume of distribution, a loading dose of at least 25–30 mg/kg over 1 h is recommended [5,9,18]. For the CI daily dose, there is no formal recommendation, leading to heterogeneous dosing. Some authors have proposed determining the dosing according to the measured creatinine renal clearance, which is correlated with vancomycin renal clearance, and further dosing guided by TDM [17,20,21].

Because these studies were very heterogeneous, the present study aimed to assess the proportion of patients with adequate vancomycin concentration after 24–48 h when applying the vancomycin dosing procedure in an institutional ICU (31 beds) over a 3-year period (intravenous bolus followed by CI). The secondary objectives were to determine the factors associated with an appropriate vancomycin serum concentration at 24–48 h, with the occurrence of acute kidney injury (AKI) and mortality.

## 2. Methods

### 2.1. Design

This study was performed according to the current French law [22] in a 31-bed French ICU [23]. It was a retrospective analysis of a prospectively designed database (2016) with data collected during a 3-year period: 1st January 2016–31st December 2018. The study was conducted in accordance with the Declaration of Helsinki, and the Institutional Review Board approved the study on November 15th 2016 (# 16.11.06). As the present study only reported the concentrations of serum vancomycin concentrations routinely collected in the ICU, the Institutional Review Board waived the patient’s informed consent. However, the patient and/or his (her) relative was verbally informed of the study and could refuse to participate. STROBE recommendations were followed for performing this observational study [24].

### 2.2. Patients

Inclusion criteria: all admitted adult patients for whom vancomycin therapy using continuous infusion had been clinically indicated by the physician in charge could be included in the present study.

Non-inclusion criteria:

- Patients <18 years of age and pregnant.
- Patients with previous renal replacement therapy (RRT) for chronic renal failure.
- Patients for whom vancomycin treatment was ongoing.
- Patients for whom vancomycin intermittent infusion was used.
- Patients requiring >14-day vancomycin therapy (such as endocarditis requiring longer treatment duration and higher plasma level).
- Patients who had previously participated to the present study.
- Patients for whom withdrawing care would be decided in the next 48 h.
- Patients not receiving intravenous dosing.
- Patients for whom a refusal was expressed.

Exclusion criteria: Patients without a measured vancomycin serum concentration.

### 2.3. Vancomycin Administration

The international and French recommendations on infections and septic shock were applied for the included patients [3,9,25,26]. For vancomycin administration (Vancomycine, Sandoz^®^, Levallois-Peret, France), a loading dose and a subsequent CI left to the physician’s decision is the current standard of practice within the unit. Dosing was adapted according to the vancomycin serum concentration, which is classically performed between 24 h (Day 2 (D2)) and 48 h (Day 3 (D3)) after vancomycin initiation and according to physician’s decision, with a serum concentration target of 20–25 mg/L. Prescription of vancomycin serum concentration measurement throughout the day was left to the discretion of the physician. Dosing adaptation modalities following a non-therapeutic vancomycin concentration were left to the discretion of the physician.

### 2.4. Measured Parameters

The following parameters were measured:

- Demographic characteristics: age, sex, height, total body weight with calculated body mass index, and previous stable serum creatinine concentration.
- Medical history, initial reason for ICU admission and Simplified Acute Physiology Score II (SAPS II) at ICU admission [27].
- The patient inclusion was defined as the day of vancomycin initiation (D1). During the inclusion period (vancomycin initiation day and 14 following days) the following data were captured: source of infection and anti-infective therapy, including type of infection, anti-infective agent(s) administered, and microbiological cultures collected.
- Clinical parameters including urine output, which was assessed at 08:00 h daily.
- The Sequential Organ Failure Assessment (SOFA) [28] and the Kidney Disease: Improving Global Outcome (KDIGO) [29] scores were calculated at inclusion (D1), daily until Day 4, and at D10 and D15.
- Requirement for vasopressor support, renal replacement therapy (RRT), mechanical ventilation, and/or sedation.
- Co-prescription of nephrotoxic drugs (e.g., aminoglycosides, diuretics, non-steroid anti-inflammatory (NSAI), iodinated contrast products).
- Biological parameters: serum creatinine concentration, calculation of creatinine clearance by chronic kidney disease-epidemiology CKD-EPI formula [30].
- Vancomycin dosing: the loading dose and the continuous infusion regimen (daily dose) and its potential alterations were recorded.
- Vancomycin assays: vancomycin serum concentrations were measured using automated immunoassays (Kinetic Interaction of Microparticles in Solution, COBAS 8000^®^, Roche Diagnostics). Blood samples for vancomycin monitoring were collected at 8:00 AM if prescribed.

The different times of measurement are shown in Table 1.

### 2.5. Objectives and Assessment Criteria

#### 2.5.1. Main Outcome

The proportion of patients with the first-available vancomycin serum concentration between 20–25 mg/L at 24 h (D2) or 48 (D3).

#### 2.5.2. Secondary Outcomes

- The proportion of patients with vancomycin serum concentration <10 mg/L at D2 or D3 (considered as a risk factor of failure and resistance emergence) [5].
- Factors associated with vancomycin serum concentration between 20–25 mg/L at D2–D3, and the factors associated with vancomycin serum concentration under 20 mg/L at D2–D3.
- Patient outcome separating (Test of Cure):
  - Clinical cure: resolution of clinical signs and symptoms compared with baseline, and no requirement for additional antibacterial treatment.
  - Clinical failure: Persistence or progression of baseline signs and symptoms after at least 2 days of treatment, consistent with active infection. When the same pathogen was found, it was considered as a relapse, whereas the presence of another pathogen was considered as a reinfection.
  - Indeterminate: Extenuating circumstances preclude classification to one of the above.
- Patient survival at ICU discharge, hospital discharge and at D28. At D28, patients with and without an appropriate serum vancomycin concentration at D2–D3 were compared.
- The occurrence of AKI (defined as a KDIGO ≥1) during the vancomycin administration, including pre-existing or beginning AKI at vancomycin initiation [29].

#### 2.5.3. Statistical Analysis

Baseline characteristics, primary outcome, and secondary outcomes were first reported. Quantitative variables were expressed as mean ± standard deviation (SD) or median (interquartile range (IQR)). Qualitative variables were expressed by their number and frequency.

In order to analyse relationships between out of target (20–25 mg/L) vancomycin serum concentration at D2–D3 (first available) and several variables of interest, logistic regression was used. Univariate analyses were first considered, and the variables for which *p*-values were under 0.20 were considered for a multivariate analysis using a stepwise selection of variables. Then, the variables with a *p*-value <0.05 in the multivariate model were considered statistically significant. The same type of analysis was performed to predict the risk factors of a vancomycin serum concentration lower than 20 mg/L at D2–D3 (first available), after exclusion of overdosed patients (>25 mg/L). Statistical analysis was performed with R 3.1.1 (The R Foundation for Statistical Computing) and SAS 9.2 (SAS Institute Inc., Cary, NC, USA).

## 3. Results

### 3.1. Study Population

**Figure 1:**
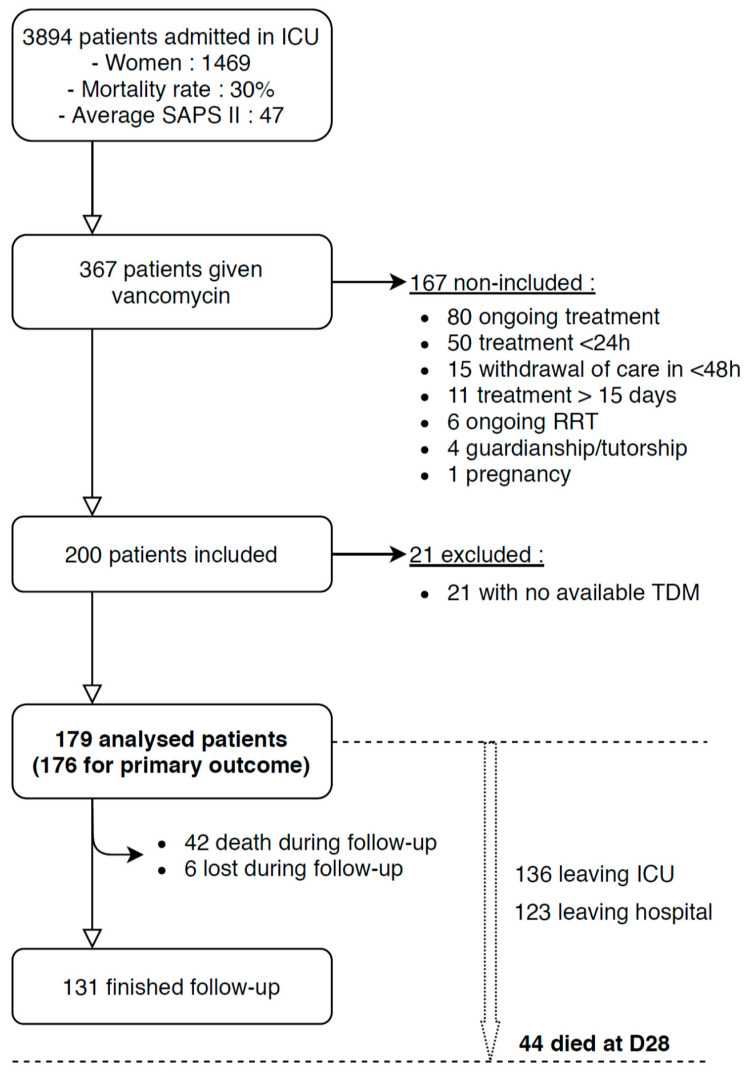
Flow chart showing patient inclusions and exclusions. TDM: therapeutic drug monitoring. Follow-up was the 15 consecutive days after vancomycin initiation. D1 was the day of vancomycin initiation.

From January 1st 2016 to December 31st 2018, 367 out of 3894 admitted ICU patients were given vancomycin. One hundred sixty seven patients did not meet inclusion criteria and 21 did not have systemic vancomycin dosing, leading to their exclusion. Therefore, 179 patients were included in this study (Figure 1).

Patient characteristics are shown in Table 2. Infection-related parameters and causative pathogens are shown in Table 3. A causative pathogen (documented infection) was isolated in 81% of patients. Intra-abdominal, bloodstream, and lung were the most frequent infection sites. Five percent of causative pathogens were MRSA. All of the pathogens had a vancomycin MIC ≤ 1.

### 3.2. Vancomycin Administration

Vancomycin was initiated 1 (0–5) day after patient admission. A loading dose of 1000 (1000–1500) mg (corresponding to 15.6 (12.5–20.8) mg/kg of total body weight) was given in 151/179 patients (84%). The median daily dose of vancomycin infusion for D1 and D2 were 2000 (1600–2000) mg (corresponding to 27.6 (21.3–31.9) mg/kg/d) and 2000 (2000–2500) mg (corresponding to 28.4 (23.8–34.8) mg/kg/d), respectively. The median duration of vancomycin administration was 4 (2–7) days. The identification and the analysis of the isolated pathogen(s) led to cessation of vancomycin infusion in 94 patients (53%).

### 3.3. Main Outcome

**Figure 2:**
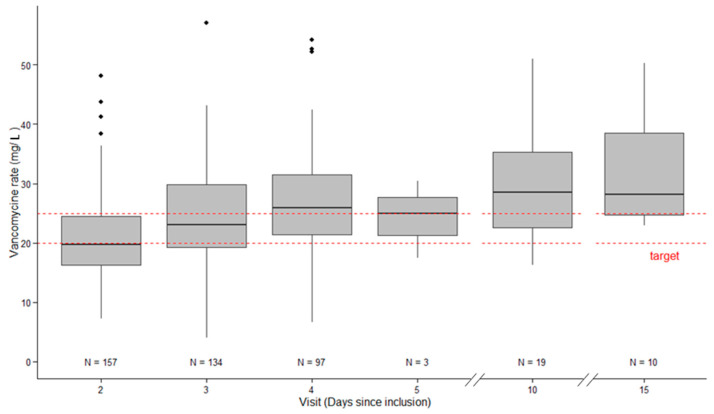
Vancomycin serum concentration over time (D2, D3, D4, D10, and D15).

Figure 2 shows the serum vancomycin concentration during its administration. At D2 or D3 (first available), the median value of serum vancomycin concentration was 19.8 (16.0–25.1) mg/L with serum vancomycin concentration between 20–25 mg/L reported in 43 patients (24%).

### 3.4. Secondary Outcomes

Serum vancomycin concentrations <20 mg/L or >25 mg/L were recorded in 89 (51%) and 44 (25%) patients, respectively. A serum vancomycin concentration <10 mg/L was recorded in 5/176 patient (3%) at D2 or D3 and never after D3. In 37 patients with RRT, 12 (32%) reached the target of serum vancomycin concentration.

In a multivariate analysis, the only parameter associated with a serum vancomycin <20 or >25 mg/L was a longer time between admission and vancomycin initiation (OR = 1.1 (1.005–1.205)) (Table 4).

Moreover, AKI at the vancomycin initiation was significantly associated with a lower incidence of serum vancomycin concentration <20 mg/L at D2–D3 (OR = 0.426 (0.199–0.912)) (Table 5). In contrast, the time between patient admission and the vancomycin initiation was associated with an increased likelihood of a serum vancomycin concentration <20 mg/L at D2–D3.

### 3.5. Patient Outcome

A clinical cure was reported in 116 patients (65%). A relapse of infection occurred in 5 patients (3%), a reinfection with another pathogen occurred in 12 patients (7%), and a clinical failure without identification was reported in 23 patients (13%). The test of cure could not be assessed in 23 patients (13%). Median duration before ICU and hospital discharge in alive patients was 6 (3–13), and 23 (14–47) days, respectively. By D28, 44 (26%) patients had died. Of the 42 patients with serum vancomycin concentration between 20 and 25 mg/L at D2–D3, 8 (19%) died by D28 whereas 37 out of 132 patients (28%) with serum vancomycin concentration <20 mg/L or >25 mg/L at D2–D3 died by D28 (*p* = 0.22).

AKI during vancomycin administration occurred in 107 (60%) patients. Among those, 49 patients (27%) required RRT after vancomycin initiation. AKI occurred in 34 out of 44 patients (77%) with serum vancomycin concentration >25 mg/L at D2–D3 leading to RRT requirement in 16 patients (36%). Of the 131 patients with serum vancomycin serum concentration <25 mg/L, only 72 (55%) developed AKI (*p* = 0.009) and 33 (25%) required RRT (*p* = 0.153).

## 4. Discussion

In the present study, a therapeutic target of serum vancomycin concentration was reached in 24% patients. A prolonged ICU stay before vancomycin initiation was associated with a higher risk of non-therapeutic exposure (i.e., <20 mg/L or >25 mg/L), especially an increased risk of subtherapeutic exposure. In patients with serum vancomycin concentration >25 mg/L, the occurrences of AKI and an RRT requirement were higher than in other included patients. A serum vancomycin concentration <10 mg/L was reported in 5/176 patient (3%) at D2 or D3 and never after D3, meaning that our dosing regimen was suitable for avoiding low vancomycin exposures.

Vancomycin remains a cornerstone for the management of patients with documented or suspected infection with MRSA or *Enterococcus faecium* even if the incidence of such resistant pathogens has decreased in Europe and in France in the last two decades [31,32]. The AUC/MIC index has the strongest association with efficacy, with a minimum threshold described around 400 [6,7], whereas AUC >700 has been associated with greater renal toxicity [33]. Because vancomycin has time-dependent pharmacodynamic characteristics, CI is used in one third of ICUs [34]. CI allows for more convenient TDM and is associated with less variability in serum vancomycin concentrations, faster target concentrations achievement, and less nephrotoxicity [11,12,13,14]. However, only one study has reported a reduction in mortality [10]. The 2009 American Society of Health-System Pharmacists (ASHP), the Infectious Diseases Society of America (IDSA), and the Society of Infectious Diseases Pharmacists (SIDP) recommend a trough target ≥15 mg/L with intermittent infusion in severe infections, whereas two French societies (Société Française d’Anesthésie et Réanimation (SFAR) and Société de Réanimation de Langue Française (SRLF)) recommend CI with a target steady-state concentration of 20 mg/L [5,9]. In the present study and in our institution, a therapeutic range of 20–25 mg/L is used. This target during CI is now recommended by the 2020 revised consensus guidelines by the American Society of Health-System Pharmacists, the Infectious Diseases Society of America, the Pediatric Infectious Diseases Society, and the Society of Infectious Diseases Pharmacists on therapeutic monitoring of vancomycin for serious methicillin-resistant *Staphylococcus aureus* infections (guidelines published after the completion of this study) [8]. Some authors have also proposed a therapeutic target of 20–30 mg/L [15,16,17]. The reason for these high targets is to increase the likelihood of achieving therapeutic exposures for methicillin-resistant *Staphylococcus* sp. with MIC >1 mg/L [21].

However, until a MIC = 1, a target >16.5 mg/L in continuous infusion (AUC/MIC > 396) could be sufficient. Considering the extremely low rate of MRSA with MIC >1 in our institution (local data not shown), an effective vancomycin concentration was possibly given in a majority of patients (Figure 2).

The present study included a similar patient population to previous studies, including >100 patients [16,21]. The mortality rate (~30%) and a documented infection incidence (~80%) were also similar to previous studies. However, the present therapeutic target was 20–25 mg/L whereas Ocampos et al., Cristallini et al., and Baptista et al. targeted a broader target range: 20–30 mg/L [16,17,21]. With our narrower therapeutic target, only 24% patients met this range. Using a therapeutic target between 20–30 mg/L, previous studies reported an appropriate vancomycin serum concentration in about 50% of patients [16,17,21], which is consistent with our results (Figure 2).

Fifty-one percent of patients were in a subtherapeutic range at D2–3, including five at a very low range (<10 mg/L). An explanation of this result could be a suboptimal use of the loading dose, performed in only 84% of the population and often insufficient (median = 15.6 mg/kg (12.5–20.8)) relative to the recommended dose (25–30 mg/kg) [5,15,35]. However, this criterion was not retrieved in this study as a risk factor of underdosing in multivariate analysis.

At Day 2, more than 30% of patients were supratherapeutic (>25 mg/L) and at D10 or D14, nearly 50% of patients still on therapy manifested concentrations >30 mg/L (Figure 2). These results provide an incentive to maintain regular vancomycin concentration monitoring until the end of the treatment. Moreover, the occurrence of new AKI and an RRT requirement was higher in patients with a serum vancomycin concentration >25 mg/L on D1 or D2 than those with vancomycin <25 mg/L (77 and 36%, respectively). However, our study is not able to determine a causality, and AKI could have happened first and led to higher vancomycin rates by accumulation. This point needs to be explored in further studies, but it is difficult to define vancomycin-caused AKI, especially in the ICU where AKI is multifactorial.

In our analysis the only factor found to be associated with non-therapeutic exposure was a prolonged duration of stay in the ICU before initiating vancomycin administration (i.e., <20 mg/L or >25 mg/L). This result has previously been reported, in a septic population [36], and could be explained by an increased volume of distribution, by overload, and renal dysfunction [18]. Alternatively, the difficulty in calculating vancomycin doses from creatinine clearance due to unstable serum creatinine concentrations or low creatinine production may explain this observation. The presence of AKI at the initiation of vancomycin therapy was associated with a lower proportion of patients with subtherapeutic concentrations, as AKI could blunt the low dosing regimen used in our study. Previous studies reported an association between creatinine clearance and vancomycin through concentration variations [16,37]. The present study did not report similar findings as we used only calculated creatinine clearance, which is poorly correlated with measured creatinine clearance in the ICU population [38,39].

Some limitations could be relevant for the present study. First, this was a single centre study and some findings may not be extrapolated well to other centres. However, the present study included more than 150 patients in a 3-year period and confirmed the main results of previous studies in terms of low achievement of therapeutic exposures as well as the importance of loading doses and assessment of renal clearance for choosing more consistently therapeutic and safer dosing regimens. Second, the present study was only focused on the therapeutic target at D2–D3 in patients in whom the physician in charge of the patient chose vancomycin administration. The extrapolation of patient outcomes should be taken with caution as the analysis of the isolated pathogen(s) led to cessation of vancomycin infusion in 53% of patients after a median CI duration of 4 days. Third, the real MIC was not determined. In our institution, nearly all MRSA MIC are <1, meaning that an effective vancomycin concentration was probably reached in more patients than currently reported (Figure 2). Determining vancomycin MIC for each MRSA could lead to a greater proportion of patients with appropriate serum concentration. In the same way, knowledge of the MIC could lead to a reduction in vancomycin dosing to avoid too high serum concentrations and side effects. Fourth, we also included patients requiring renal replacement therapy, who likely to have different dosing requirements to other patients [40]. Fifth, the occurrence of KDIGO = 1 defined a vancomycin-associated AKI that is different from vancomycin-induced AKI, as many confounding factors could lead to AKI in such circumstances, explaining the high rate of AKI in the present study. Given this, it is perhaps unsurprising that we observed a higher rate of nephrotoxicity than previously reported [41].

## 5. Conclusions

The present study confirmed the major findings that have been recently reported in different studies: (1) The importance of loading dose: in the present study, a loading dose of 15.6 (12.5–20.8) mg/kg led to a nearly 50% vancomycin serum concentration at D2. Therefore, a loading of 25–30 mg/kg should be encouraged. (2) The importance of measuring creatinine clearance for anticipating the need of increased infusion rate. (3) The need of TDM, because of persistent difficulties to predict vancomycin serum concentration in ICU patients, and for avoiding a too high vancomycin serum concentration that could impair renal function.

## Figures and Tables

**Table 1:** Time of measurement of the different studied parameters.

Collected Data	D1	D2	D3	D4	D5	D6	D7	D8	D9	D10	D11	D12	D13	D14	D15
Weight	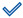	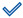	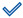	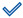	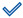	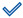	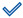	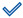	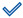	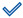	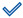	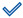	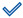	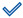	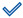
SOFA-score	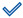	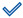	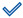	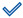						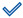					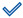
Serum creatinine	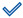	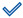	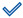	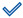	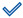	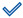	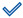	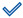	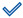	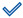	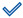	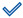	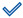	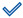	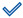
GFR by CKD-EPI	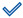	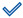	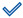	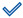	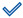	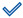	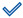	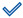	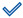	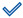	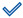	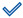	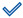	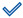	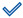
AKI by KDIGO-stage	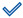	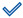	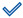	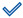						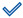					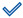
Vancomycin loading dose *	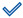	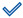	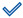	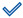						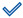					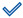
Vancomycin daily dose	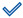	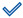	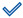	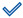	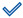	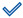	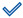	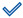	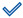	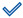	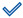	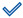	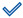	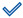	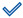
Dose adaptation	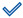	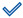	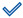	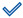	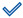	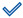	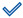	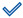	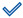	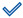	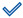	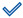	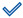	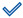	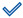
Hemodynamic support	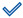	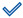	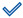	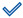						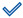					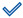
Mechanical ventilation	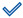	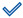	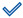	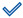						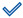					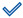
Sedation	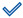	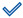	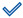	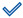						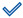					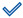
Dialysis	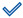	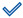	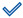	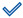						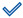					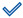
Nephrotoxic comedication	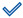	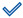	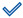	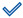						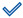					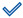
Iodine contrast products	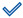	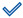	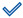	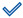						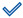					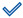

* Vancomycin loading doses after D1 were generally used to quickly increase a very low vancomycin concentration and were left to the discretion of the physician. AKI: acute kidney injury; GFR: glomerular filtration rate; KDIGO: kidney disease: improving global outcome [29]; SOFA: sequential organ failure assessment [28]; CKD-EPI: chronic kidney disease-epidemiology [30]; D1 = day 1, i.e., vancomycin initiation day; D2 and following are the subsequent days.

**Table 2:** Patient characteristics.

Characteristics	Analysed Patients	Value
Admission, median (IQ 25–75):		
- Age (years)	179	67 (59–75)
- Women, n (%)	179	60 (34)
- SAPS II score	179	48 (36–57)
- Weight (kg)	179	74 (63–86)
- Height (m)	179	1.70 (1.62–1.75)
- Body Mass Index (kg/m^2^)	179	25.7 (22.3–29.1)
- Baseline Creatinine (µmol/L) *	177	68 (59–86)
- Creatinine (µmol/L)	179	104 (72–162)
- Creatinine Clearance by CKD-EPI (mL/min)	178	62 (34–88)
- Albumin (Admission)(g/L)	170	28 (23–32)
- ICU length of stay before vancomycin initiation (day)	179	1 (0–5)
Comorbidities, n (%) ^Ɨ^	179	
- Diabetes mellitus		34 (19)
- Coronaropathy		17(10)
- Cardiac insufficiency		13 (7)
- Peripheral arterial disease		29 (16)
- Cancer		11(6)
- Liver cirrhosis		8(4)
- High blood pressure		55 (30)
- Single kidney		5(3)
- Renal transplantation		0 (0)
Treatment at admission, n (%)	179	
- Conversion enzyme inhibitor		36 (20)
- Angiotensin II receptor inhibitor		17 (10)
- Diuretics		38 (20)
- Non-steroid anti-inflammatory		16 (9)
- Chemotherapy ᵋ		8 (4)
- Others		22 (12)
Admission type, n (%)		
- Sepsis or septic shock, n (%)	179	110 (61)
Vancomycin therapy, median (IQ 25;75) ᵠ		
- loading dose, mg	151	1000 (1000–1500)
- loading dose, mg/kg	151	15.6 (12.5;20.8)
- Day 1 daily dose, mg	178	2000 (1600–2000)
- Day 1 daily dose, mg/kg	178	27.5 (21.3–31.9)
- Day 2 daily dose, mg	177	2000 (2000–2500)
- Day 2 daily dose, mg/kg	177	28.4 (23.8–34.8)
Other nephrotoxic drugs during follow-up, n (%)	179	172 (96%)
- Aminoglycosides	179	142 (79%)
Organ failure, n (%) ᵡ		
- Mechanical ventilation	179	152(85)
- Renal replacement therapy	179	49 (27)
- Vasoactive drugs	179	110(61)
Glomerular filtration, n (%)		
- AKI during vancomycin administration ᶷ	179	107 (60)
- Augmented renal clearance at Day 1 or Day 2 ᶿ	179	7(4)
Outcome		
- Day 28 Mortality, n (%)	178	46 (26)
- Length of stay, ICU, (d) ‡	136	6 (3-13)
- Length of stay, hospital, (d) ‡	123	23 (14–47)

SAPS: simplified acute physiological score II [27]; CKD-EPI: chronic kidney disease-epidemiology [30]; ICU: intensive care unit; AKI: acute kidney injury. * Last creatinine measured in a previous stay or registered baseline creatinine or age, sex, and race adapted calculation if not available. Ɨ As registered in patient’s file. ᵋ Chemotherapy for any cancer. ᵠ Day 1 was the day of vancomycin initiation, Day 2 was the day after. ᵡ during follow-up. ᶷ according to KDIGO stage. ᶿ Augmented renal clearance was defined by a renal clearance >130 mL/min; ‡ Among survivor.

**Table 3:** Infection and causative pathogen patterns.

Infection Characteristics, n (%)	Analysed Patients	Value
Documented infection site:	143	
- Intra-abdominal		53 (37)
- Bloodstream		29 (20)
- Lung		25 (17)
- Urinary tract		15 (10)
- Skin		9 (6)
- Bone		9 (6)
- Brain meninges		3 (2)
Documented causative pathogen	143	
- MRSA		7 (5)
- MSSA		21 (15)
- *Coagulase negative Staphylococcus*		21 (15)
- *Enterococcus faecalis*		22 (15)
- *Enteroccocus faecium*		23 (16)
- Others GPC		15 (10)
- Enterobacterales		71 (50)
- *Pseudomonas aeruginosa*/*Acinetobacter baumannii*		18 (13)
- Others GNB		10 (7)
- Anaerobes		13 (9)
- Yeast		17 (12)
- Others		2 (1)
Polymicrobial infection site	143	61 (43)

MRSA: Meticillin-resistant *Staphylococcus aureus*; MSSA: Meticillin-sensitive *Staphylococcus aureus*; GPC: Gram-positive cocci; GNB: Gram-negative bacilli.

**Table 4:** Multivariate analysis for risk factors of being out of target.

Variables	Univariate Analysis				Multivariate Analysis			
	Odds-Ratio	95% CI	*p*-Value		Odds-Ratio	95% CI	*p*-Value	
Age	0.992	0.967	1.018	0.5580				
Sex: Woman vs. Man	1.864	0.845	4.110	**0.1230**				
SOFA Score	0.963	0.882	1.050	0.3923				
Dialysis at D1 or D2	0.598	0.270	1.325	0.2051				
AKI D1 or D2	0.625	0.311	1.258	**0.1879**				
Augmented renal clearance at D1 or D2	1.984	0.232	16.959	0.5313				
Nephrotoxic co-medication at D1 or D2	1.138	0.343	3.776	0.8333				
Obesity	0.811	0.365	1.804	0.6078				
Albuminemia	1.014	0.972	1.059	0.5137				
Length of stay in ICU before vancomycin initiation (day)	1.100	1.005	1.205	**0.0395**	1.100	1.005	1.205	**0.0395**
Loading dose (mg/kg)	0.990	0.934	1.050	0.7446				
Daily dose (mg/kg)	0.997	0.963	1.033	0.8765				

AKI: acute kidney injury as defined by KDIGO stages; D1: day of vancomycin initiation, D2: the day after; ICU: intensive care unit.

**Table 5:** Multivariate analysis for risk factors of being under the target.

Variables	Univariate Analysis				Multivariate Analysis			
	Odds-Ratio	95% CI		*p*-Value	Odds-Ratio	95% CI		*p*-Value
Age	0.986	0.957	1.015	0.3331				
Sex: Woman vs. Man	1.763	0.768	4.048	**0.1810**				
SOFA Score	0.922	0.838	1.014	**0.0956**				
Dialysis at D1 or D2	0.442	0.182	1.075	**0.0717**				
AKI D1 or D2	0.404	0.192	0.852	**0.0173**	0.426	0.199	0.912	**0.0280**
Augmented renal clearance at D1 or D2	3.036	0.354	26.042	0.3112				
Nephrotoxic co-medication at D1 or D2	1.201	0.332	4.348	0.7797				
Obesity	0.687	0.289	1.632	0.3948				
Albuminemia	1.011	0.952	1.073	0.7321				
Length of stay in ICU before vancomycin initiation (day)	1.112	1.007	1.227	**0.0350**	1.102	0.998	1.217	**0.0539**
Loading dose (mg/kg)	0.981	0.922	1.043	0.5327				
Daily dose (mg/kg)	0.984	0.944	1.025	0.4321				

AKI: acute kidney injury as defined by KDIGO stages; D1: day of vancomycin initiation, D2: the day after; ICU: intensive care unit.
